# The influence of amyloid on tau deposition in clinically obese participants

**DOI:** 10.1002/alz.092313

**Published:** 2025-01-09

**Authors:** Yang Yang Yu, Joseph Nowell, Paul Edison

**Affiliations:** ^1^ Imperial College London, London UK

## Abstract

**Background:**

Obesity is a prevalent comorbid condition that doubles the risk of Alzheimer’s disease. The proposed mechanisms underlying neuronal damage involve chronic low‐grade inflammation, the production of reactive oxidative species, and altered brain glucose metabolism. In Alzheimer’s disease, amyloid is thought to influence tau deposition and glucose metabolism. We explore the impact of amyloid on tau pathology in individuals with obesity.

**Method:**

We processed 267 PET scans (18F‐flortaucipir) from 267 participants with a BMI ≥30 (n=116) and those without obesity (n=151) in the Alzheimer’s Disease Neuroimaging Initiative (ADNI) dataset to compare tau pathology. Amyloid status was determined from the ADNI database using predefined cerebellum positivity thresholds (SUVR of 1.08 for ^18^Florbetaben and 1.11 for ^18^florbetapir). Independent t‐tests and multivariate analysis were employed in subjects with obesity versus those without, in global cortex and specific regions of interests in relation to Alzheimer’s. Amyloid positive and negative subjects were evaluated separately within different cognitive subgroups, namely those with mild cognitive impaired and those who were cognitively normal.

**Result:**

Independent t‐tests were conducted in the obese group with normal cognition and negative amyloid status, revealing higher tau uptake in the parietal lobe and anterior cingulate compared to non‐obese subjects. Multivariate analysis, controlling for age and APOE‐ε4, further confirmed the significance of these findings in these brain regions. This pattern was absent in obese subjects with positive amyloid status and in those with mild cognitive impairment, regardless of amyloid status being positive or negative.

**Conclusion:**

Tauopathy manifests in the obese brain independently of amyloid deposition, even preceding cognitive impairment. These findings suggest that amyloid‐targeting therapies may be less effective in obese patients.

